# Venous Thromboembolism – Current Diagnostic and Treatment Modalities

**DOI:** 10.3889/oamjms.2016.087

**Published:** 2016-08-24

**Authors:** Marijan Bosevski, Elizabeta Srbinovska-Kostovska

**Affiliations:** *University Cardiology Clinic, Faculty of Medicine, Ss Cyril and Methodius University of Skopje, Skopje, Republic of Macedonia*

**Keywords:** venous thromboembolism, pulmonary embolism, deep venous thrombosis treatment, novel oral anticoagulants

## Abstract

**BACKGROUND:**

Pulmonary embolism and deep venous thrombosis, known as venous thromboembolism (VTE), are associated with a high proportion of morbidity and mortality.

**AIM:**

Aim of this review is to emphasise current diagnostic and therapeutic modalities for VTE.

**RESULTS:**

No differences have been noticed in European and American guidelines in diagnostic approach of this disorder. Today there is enough clinical information for the use of heparin (either, unfractionated or low molecular) and vitamin K antagonists in the treatment of acute and chronic phases of VTE. Novel oral anticoagulants seem to have some advantages in the treatment of this disorder. Rivaroxaban has been approved widespread, for use as a single-drug approach of VTE.

**CONCLUSION:**

Both guidelines are almost similar and good basis for evidence-based treatment of this disorder.

## Introduction

Pulmonary thromboembolism (PE) is a disorder of the pulmonary circulation because of the presence of thrombi. Thrombi are usually formed in the venous circulation (deep vein thrombosis or DVT). These two entities are known by the common term: venous thromboembolism.

The importance of VTE comes from its association with a high proportion of morbidity and mortality [1]. This disorder is often asymptomatic, misdiagnosed, unrecognised and untreated.

### Diagnosis

The diagnosis is established on the basis of clinical presentation and risk factors present. Clinical signs and symptoms are often nonspecific, for which everyVTE is hardly familiar. The most common symptoms are loss of consciousness (due to a relative lack of oxygen supply to the brain), dyspnea or tachypnea (because of respiratory decline in vital capacity, atelectasis and vasoconstriction), chest pain (RH=right heart ischemia, pleural effusion), hemoptysis, (alveolar hemorrhage), and high temperature and cough (additional infection). Symptoms are present with the obstruction of more than 50 % of pulmonary circulation. Sudden cardiac death occurs in obstruction of the pulmonary arteries. This, in turn, produces large pulmonary peripheral resistance, triggers right heart failure and systemic hypotension, while isolated segmental and supsegmental VTE do not initiate these dramatic clinical symptoms [2].

The clinical probability for PE is based on the existence of risk factors and their significance. There is not a universal cutpoint for D-dimer, it is assayed dependent D-dimer test is used as a test to exclude PE.

Doppler ultrasound of deep veins of the legs is a diagnostic test for the presence of VTE. Ventilation-perfusion lung scan is a proven diagnostic test for suspected PE with low sensitivity.

Echocardiography has a limited role in PE. However, it is used, where transfer for definitive imaging is not possible and thrombolysis is being considered. CT angiography (multislice) is a method of choice for the diagnosis of PE by direct evidence of a clot, breakdown charge pulmonary arterial branch. Pulmonary angiography by Seldinger is the gold standard, although is used less often. All mentioned diagnostic tools are part of the diagnostic algorithm for PE. Echocardiography is sufficient to diagnose PE in high-risk patients. CT angiography is completed when the patient is stabilised, or when it is available.

Differential diagnoses are noted as acute myocardial infarction, myocarditis or pneumothorax or specific conditions of right heart failure. Diagnosis of deep vein thrombosis is based on to the vascular ultrasound examinations. Vein ultrasound detects noncompresibiily of affected vein, loss of respiratory phasic signal and visualisation of thrombus [3].

### Treatment

Rapid initiation of treatment is most important even when there is a suspicion of VTE [4, 5].

Acute treatment of patients with PE (without high risk) consists of the use of heparin (unfractionated or low-molecular) or fondaparinux. During this phase, a laboratory with blood tests is required, along with the values of activated thromboplastin or kaolin cefalin’s time. Both heparins are known to be safe and as effective. In comparison with low-molecular-weight heparin, initial therapy with unfractionated heparin was associated with higher mortality and a higher rate of fatal pulmonary embolism in patients. The treatment continues with oral vitamin K anticoagulants (Acenocoumarol) in accordance with appropriate scheme value of INR (International proportion value to be between 2 and 3). Alternatively, treatment with Dabigatran or Rivaroxaban is equivalent in the long term phase of treatment.

High-risk PE is an indication, however, for quick treatment with fibrinolytic therapy.

Novel oral anticoagulants seem to have some advantages in the treatment of this disorder. Current Cohen’s meta-analysis indicates that the NOACs have a clinical benefit over conventional therapy while compared to their relative differences in bleeding profile.

Rivaroxaban has been approved for widespread use as a single-drug approach of VTE. This drug is not inferior in the treatment of PE (not high risk) in its acute phase. In patients who had acute symptomatic proximal DVT, without symptomatic PE, Rivaroxaban showed: Non-inferiority for efficacy when compared with treatment regimen LMWH/VKA, same safety outcomes (irrespective of age, gender and body weight, renal function or presence of cancer) and no evidence of hepatic toxicity.

But, until today, there is no statement on which NOACs might be more beneficial (or have greater evidence) versus standard coagulation in the acute phase of VTE [6]. Rivaroxaban is approved as a medication for all indications in the treatment and prevention of venous thromboembolism, the prevention of VTE in patients undergoing major orthopaedic surgery, and for nonvalvular atrial fibrillation in order to prevent systemic peripheral embolism, including stroke. These recommendations are contained in the current guidelines of the European Society of Cardiology and the American Lung Physicians’ Association. These noted here are based on EINSTEIN studies’ results.

A comparison of VKA antagonist, NOAC and direct thrombin inhibitors is given in Table 1.

**Table 1:**
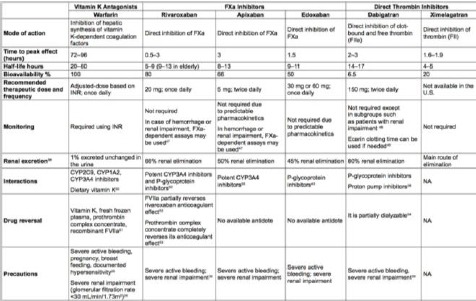
Characteristics of VKA antagonist, NOAC and direct thrombin inhibitors [7]

VKA have some disadvantages as narrow therapeutic window, regular coagulation monitoring and dose adjustment, lack of staying in therapeutic range (INR 2-3) for all pts and consequent increase in the risk of thromboembolic events or side effects – bleeding, significant inter - and intra - individual variations in the response due to: numerous interactions with food and medicine and complex pharmacokinetics and pharmacodynamics.

Because of the foreseeable pharmacology and broad therapeutic window, Rivaroxaban is the drug of choice and replaced other anticoagulants.

The minimum duration of anticoagulation for VTE is 3 months (m); Till today, there is no evidence to suggest 6 m is more effective than 3 m. Duration of treatment of patients with transient risk factor is up to 6 months. Long-term anticoagulant therapy is common in patients without apparent risk factors and those with proven thrombophilia (hypercoagulability). It is aimed for prevention of fatal and non- fatal recurrent VTE events taking into account the risk of bleeding.

Due to CHEST Guidelines, for VTE long-term anticoagulant therapy, rivaroxaban and another NOAC is suggested one, (Grade 2B), over vitamin K antagonist (VKA) therapy, and VKA therapy instead of low-molecular-weight heparin (LMWH; Grade 2C). For VTE and cancer, we suggest LMWH over VKA (Grade 2B), Rivaroxaban and other NOACS (Grade 2C) [8]. European Guidelines on pulmonary embolism is an evidence-based document for VTE with equivalent evidence for its treatment. A Macedonian recommendation for treatment of PE is an evidence-based document, coming from European Guideline.

*Prognosis* of venous thromboembolism depends on of disease severity, aetiology and timing from diagnosis to initiation of therapy. VTE pts with appropriate clot lysis have a better survival rate. Rivaroxaban, as a novel anticoagulant is approved for the acute and chronic phase of treatment of VTE. Patients’ adherence according to CHEST guidelines was high and resulted in low rates of recurrent VTE and bleeding. The risk of VTE recurrence after discontinuation of therapy is primarily determined by two factors: whether the acute episode was treated effectively and internal risks of a new episode of VTE. All patients with present pulmonary hypertension have impaired quality of life and worse survival [9].

In conclusion, pulmonary embolism and deep venous thrombosis, also known as venous thromboembolism (VTE), are associated with the high proportion of morbidity and mortality. Current guidelines propose Computer Tomography angiography, Echocardiography, Venous ultrasound and D-dimmers test for diagnosis of VTE.

Early initiation of treatment of VTE is a crucial one. Rivaroxaban and other novel oral anticoagulant are effective in a treatment of venous thromboembolism, as traditional anticoagulants are. In that way, both guidelines are almost similar and good basis for evidence-based treatment of this disorder.

## References

[1] Monreal M, Mahé I, Bura-Riviere A, Prandoni P, Verhamme P, Brenner B, Wells PS, Di Micco P, Bertoletti L (2015). Pulmonary embolism: Epidemiology and registries. Presse Med.

[2] Skhiri M, Hunt SA, Denault AY, Haddad F (2010). Evidence-Based Managеmеnt of Right Heart Failure: a Systematic Review of an Empiric Field. Rev Esp Cardiol.

[3] Jiménez D, de Miguel-Díez J, Guijarro R, Trujillo-Santos J, Otero R, Barba R, Muriel A, Meyer G, Yusen RD, Monreal M (2016). RIETE Investigators Trends in the Management and Outcomes of Acute Pulmonary Embolism: Analysis From the RIETE Registry. J Am Coll Cardiol.

[4] Kearon C, Akl EA, Ornelas J, Blaivas A, Jimenez D, Bounameaux H, Huisman M, King CS, Morris TA, Sood N, Stevens SM, Vintch JR, Wells P, Woller SC, Moores L (2016). Antithrombotic Therapy for VTE Disease: CHEST Guideline and Expert Panel Report. Chest.

[5] SEC Working Group for the ESC 2014 Guidelines on the Diagnosis and Management of Acute Pulmonary Embolism; Expert Reviewers for the ESC 2014 Guidelines on the Diagnosis and Management of Acute Pulmonary Embolism;SEC Clinical Practice Guidelines Committee (2015). Comments on the 2014 ESC Guidelines on the diagnosis and management of acute pulmonary embolism. Rev Esp Cardiol (Engl Ed).

[6] Moliterno DJ, Kristensen SD, De Caterina R (2013). Therapeutic advances in thrombosis.

[7] DeLoughery G (2015). Hemostasis and Thrombosis.

[8] Toth PP (2016). Considerations for long-term anticoagulant therapy in patients with venous thromboembolism in the novel oral anticoagulant era. Vasc Health Risk Manag.

[9] Martinez K, Kosirog E, Billups SJ, Clark NP, Delate T, Witt DM (2015). Clinical outcomes and adherence to guideline recommendations during the initial treatment of acute venous thromboembolism. Ann Pharmacother.

